# Dr. Schenk on Croup

**Published:** 1810-12

**Authors:** 


### Dr. Schenk on Croup.

CROUP is a disease so rapid in progress and fatal in termination, that it affords little time to consider the mode of treatment proper to be pursued; our practice must be prompt and decided. Yet we find practitioners vary considerably in opinion respecting the means of cure in this complaint. Some insist upon copious bleeding as the only chance of saving the patient, whilst others trust entirely to mercury.

Dr. Schenk, of Siegen, has stated in one of the German Journals an interesting account of his experience in this malady, of which, in the course of nine years, he has had occasion to treat twenty-two cases. The first was a child five years old, who had just recovered from the measles, but being exposed too soon to the air of an open window, was attacked with hoarseness, which on the following day increased, and was accompanied by a cough. Dr. Schenk considered it as a metastasis of the measles on the bronchiæ, applied a blister, and gave camphor and Aq. Ammon. Acet. The third day, the cough had a hollow sound, as if proceeding from a tun; the child complained of a stoppage in the throat which rendered respiration difficult; the voice was extremely hoarse; and at intervals he struggled with suffocation. The disease was was now apparent, but the remedies were applied too late, and the patient died on the evening of the third day.

This catastrophe left an indelible impression on the author's memory, and being called some month's afterwards to visit two children who were affected with what was termed a violent catarrh, on the third day of the attack he had no difficulty in undeceiving the parents respecting the nature of the malady which affected their children, who both died in the evening of that same day.

Upon opening the bodies, a lymphatic membrane was observed lining the bronchial vessels throughout their extent; in some parts, it adhered so closely, as scarcely to admit of being separated. This convinced the author that an opening into the trachea would not have preserved life.

The same winter (1800) he attended two girls of six and four years of age, in this complaint. He immediately pursued the practice of Lentin, which chiefly consists in the application of leeches, and the use of mercury, both externally and internally. On the second day the eldest was out of danger, and the youngest better. On the third, this also was considered to be safe. The mercury was discontinued, and a decoction of senega, with pectoral elixir, and syrup of gum ammoniac, was given. On the fourth day, the child was cheerful and going on well, though still occasionally coughing as in a common cold, without exciting any suspicion of danger. On the fifth day the complaint returned, and the child died on the sixth, although the use of mercury was resumed. Dr. Schenk attributed the relapse to this remedy being discontinued too soon. He was the more confirmed in this opinion, when, in the following spring, he succeeded in curing four children in the second day of the complaint by the internal and external administration of mercury, and in three of these cases, that was the only remedy employed, the leeches, so strongly recommended by Lentin, not being applied.

Since that period he has treated fourteen cases of Croup, and of these, eight recovered by the use of mercury alone; in the remaining six, the disease was too far advanced, Dr. Schenk not being called in till the third, or even fourth day.

